# TRIM26-mediated regulation of TRAF6 ubiquitination enhances host immune response during *Toxoplasma gondii* infection

**DOI:** 10.1007/s00018-026-06088-2

**Published:** 2026-02-06

**Authors:** Xudian An, Xiaoyan Zhao, Ting Zeng, Huijie Qiu, Lingyu Li, Min Gao, Shumin Gao, Daiang Liu, Chunxue Zhou, Bing Han, Huaiyu Zhou

**Affiliations:** Department of Pathogen Biology, School of Basic Medical Sciences, Cheeloo College of Medicine, Shandong University, Jinan, Shandong Province 250012 PR China

**Keywords:** *Toxoplasma gondii*, TRIM26, TRAF6, Ubiquitination, Immune response, Inflammation

## Abstract

*Toxoplasma gondii* is a parasitic protozoan that poses a significant threat to both human and livestock. Currently, effective control measures remain elusive, largely due to the unclear mechanisms underlying *T. gondii* infection and the host immune response. This study aims to elucidate the role of tripartite motif-containing 26 (TRIM26) in modulating the host immune response to *T. gondii* via regulating tumor necrosis factor receptor-associated factor 6 (TRAF6). Our findings revealed that *T. gondii* infection significantly upregulates TRIM26 expression in murine macrophages. Notably, TRIM26 acts as a novel regulator of TRAF6 by reducing K48-linked polyubiquitination of TRAF6, which in turn promotes the expression of downstream cytokines. This indicates that TRIM26 plays a critical role in the host immune response against *T. gondii*. Furthermore, in vivo investigations demonstrated that TRIM26 expression is upregulated during *T. gondii* infection. Additionally, *Trim26*^*−/−*^ mice exhibited significantly reduced proportions of macrophages, inflammatory monocytes in ascites, and T cells in the spleen following infection. These knockout mice also exhibited reduced survival rates, decreased levels of IL-12, IFN-γ, and TNF-α, more severe organ pathological damage, and markedly higher parasite burdens compared to wild-type mice. Collectively, this study highlights the role of TRIM26 in regulating TRAF6 ubiquitination, enriches our understanding of the mechanism of TRAF6 modification. Additionally, it elucidates the significance of TRIM26 in the host immune response to *T. gondii*, and providing new insights and potential targets for the prevention and treatment of toxoplasmosis.

**Supplementary Information:**

The online version contains supplementary material available at 10.1007/s00018-026-06088-2.

## Introduction

*Toxoplasma gondii* is an obligate intracellular parasite that can invade any nucleated cell in all warm-blooded animals, including humans. It is globally distributed, with approximately one-third of the world’s population harboring chronic infection [1, 2]. This opportunistic pathogen causes toxoplasmosis, which can be life-threatening in immunocompromised individuals such as those with AIDS, cancer, and organ transplant recipients. In contrast, the infection is generally asymptomatic in most immunocompetent individuals. Human infection is primarily acquired by ingestion of cysts in undercooked meat, raw vegetables, contaminated water and other sources [3]. The frontline treatment for acute infection involves a combination of pyrimethamine and sulfadiazine [4]. Unfortunately, no effective vaccine is currently available to prevent infection in humans. Therefore, it is imperative to elucidate the immune response to *T. gondii* infection. A comprehensive understanding of host anti-*T. gondii* immunity is crucial for identifying new approaches or targets for combating *T. gondii* infection.

The innate immune system serves as the first line of defense against invading parasites, with pathogen recognition being crucial in the immune response to *T. gondii* [5]. This early immune response is mediated by pathogen recognition receptors, particularly toll-like receptors (TLRs) [6–9]. The activation of myeloid differentiation primary-response protein 88 (MyD88), dependent on TLRs, triggers strong T helper 1 (Th1) cell responses, characterized by the production of IL-12 and IFN-γ, is essential for clearing *T. gondii* [10]. It is universally acknowledged that the elimination of parasites requires pro-inflammatory cytokine IL-12, which stimulates natural killer (NK) cells, as well as the CD4^+^ and CD8^+^ T cells, to generate IFN-γ. Both acute and chronic infections stimulate NK cell cytotoxicity, contributing to the destruction of parasite-infected cells [11]. IL-12 can heighten the NK cells and T cells cytotoxicity and drives Th1 immune response by promoting Th1 cells differentiation [12]. Additionally, IL-12 and IFN-γ are essential for the maintenance of inflammatory DCs at the site of infection with *T. gondii* [13]. IFN-γ is a major effector molecule in the host’s resistance to *T. gondii* and plays a vital role in activating antimicrobial pathways that limit parasite replication [14–17]. The innate immune system determines the outcome of toxoplasmosis, and strengthening this response is beneficial to eradicate *T. gondii* and prevent chronic infection.

Tumor necrosis factor receptor-associated factor 6 (TRAF6) is an E3 ubiquitin ligase and essential adaptor protein that triggers the canonical NF-κB pathway and up-regulates IL-12 cytokines expression during *T. gondii* infection [18, 19]. Host cell TLRs recognize parasite-associated molecules and recruit downstream MyD88, which phosphorylates IRAK1 and IRAK4, subsequently activating TRAF6 [20]. TRAF6 then promotes auto-ubiquitination through K63-linked polyubiquitin chains, leading to the activation of the TAK1, TAB1 and TAB2 complex [21–23]. This complex subsequently induces the phosphorylation of IKK complex, resulting in the activation and nucleus translocation of NF-κB, where it initiates the expression of pro-inflammatory cytokines [24].

Additionally, TRAF6 can directly target the parasitophorous vacuole and enhance ubiquitination to catalyze its destruction [25]. Given TRAF6’s role in the innate immune system, it is a strategic target for invading pathogens to manipulate the host immune response. For instance, the *Avibirnavirus* VP3 protein interacts with the zinc finger domain of TRAF6 to mediate the K11 and K33-linked ubiquitination, facilitating virus nucleus accumulation to promote itself proliferation [26]. In human fibroblasts, *T. gondii* effector GRA15 recruits TRAF6 to the PV membrane, leading to lysosomal degradation of the vacuole. This interaction promotes the transformation of acute infection into chronic infection, benefiting the prolonged survival of *T. gondii* [19]. Therefore, identifying regulatory proteins that modulate TRAF6, enriching the regulatory network, and elucidating new mechanism of TRAF6 modification are of great significance for understanding the interaction between *T. gondii* and the host.

This study aimed to investigate whether novel regulatory factors regulate the fundamental functions of TRAF6. We identified TRIM26 as a TRAF6-interacting factor that modulates TRAF6 ubiquitination by reducing K48-linked chains. The absence of TRIM26 led to decreased phosphorylation of several adaptor molecules and attenuated the activation of the TRAF6-dependent NF-κB signaling pathway, reducing inflammatory cytokines production, particularly IL-12 and IFN-γ. We also demonstrated the significant role of TRIM26 in the immune response induced by *T. gondii.* These studies clarified a novel mechanism for TRAF6 ubiquitination modulation and further explored the critical impact of TRIM26 in toxoplasmosis.

## Materials and methods

### Mice

Female C57BL/6 mice (wild type) were purchased from the Animal Center of Shandong University, People’s Republic of China. *Trim26*^*−/−*^ mice were generously provided by Professor Chengjiang Gao from the Department of Immunology, School of Basic Medical Sciences, Shandong University. All mice were housed under specific pathogen-free conditions with appropriate temperature and ventilation controls. All animal experiments were performed in strict accordance with the Animal Ethics Procedures and Guidelines of the People’s Republic of China, and approved by the Institutional Animal Care and Use Committee of Shandong University under Contract (Permit No. ECSBMSSDU2019-2–006).

### Cells and parasites

HFF cells, BV2 cells, and HEK293T cells were obtained from the Cell Bank of the Chinese Academy of Sciences (Shanghai, China). These cells were cultured in Dulbecco’s Modified Eagle medium (DMEM, Cellmax, China), supplemented with 10% fetal bovine serum (FBS, Cellmax, China), and 1% penicillin/streptomycin (Solarbio, China). Bone marrow-derived macrophages (BMDMs) were isolated from the femoral and tibial bone marrow of *Trim26*^*−/−*^ and WT mice, and differentiated for 7 days in DMEM medium supplemented with 10% FBS, 1% penicillin/streptomycin, and 20 ng/ml M-CSF. All cells were maintained in a humidified incubator at 37 °C with 5% CO_2_. *T. gondii* tachyzoites of the type Ⅰ RH strain and type Ⅱ ME49 strain were maintained in our laboratory using immortalized HFF cells.

### Western blotting

Cells were stimulated as indicated and lysed on ice using RIPA buffer (Beyotime, China) supplemented with fresh protease inhibitor (Bimake, Texas, USA). Protein samples were diluted and heated at 95 ℃ for 10 min. Equal amounts of protein samples were separated using SDS-PAGE and transferred onto polyvinylidene difluoride (PVDF) membranes (Millipore, Massachusetts, USA). Membranes were blocked with Tris-buffered saline supplemented with 0.05% Tween (Solarbio, China) and 5% skimmed milk for 2 h at room temperature, followed by incubation with specific antibody overnight at 4 °C. Antibodies used were as follows: rabbit anti-TRAF6 (ab137452, Abcam), mouse anti-TRIM26 (sc-393832, Santa Cruz Biotechnology), anti-p65 (6956), anti-p-p65 (3033), anti-IκBα (4812), anti-p-IκBα (2859), anti-TAK1 (4505), anti-p-TAK1 (9339), anti-IKKα/β, and anti-p-IKKα/β (2697) from Cell Signaling Technology, rabbit anti-HA (51064-2-AP), anti-β-Actin (66009-1) and anti-GAPDH (60004-1) from Proteintech. Secondary antibodies (goat anti-rabbit, AB0101; goat anti-mouse, AB0102) were from Abways; Anti-Flag (R20008) was from Abmart, and mouse anti-Myc (TA150121) was from Origene. Blots were then washed and probed with the respective horseradish peroxidase-conjugated secondary antibody for 1 h at room temperature. Images were developed using ECL detection reagents (Millipore, Massachusetts, USA) and captured on Tanon 5200 Chemiluminescent Imaging System (Tannon, China).

### RNA isolation and real-time quantitative PCR

Total RNA was extracted using the RNA Easy Fast Tissue/Cell kit (TIANGEN, DP451) according to the manufacturer’s instructions. RNA was reverse transcribed to cDNA using reverse transcriptase (Vazyme, R323-01), and cDNA was used to assess gene expression using the SYBR Green method (Vazyme, Q712). Data were analyzed using Bio-Rad CFX Maestro software (Bio-Rad, USA). Relative gene expression levels were normalized to β-actin as the control. Primer sequences are listed in Supplementary Table S1.

### Enzyme-linked immunosorbent assay (ELISA)

Supernatants from cell cultures and serum were collected in sterile tubes and centrifuged at 2–8 ℃ for approximately 20 min at 2,000 rpm-3,000 rpm. The concentrations of IL-12, IFN-γ, TNF-α, IL-6, and IL-1β were measured using ELISA kits (Dakewe Biotech, Shenzhen, China) according to the manufacturer’s instructions.

### Plasmids and transfection

The TRAF6-HA and TRIM26-Flag plasmids were constructed by inserting the full - length coding sequence of murine TRAF6 and TRIM26 gene into the pcDNA3.1(+) vector. The ubiquitin plasmids (WT, K6, K11, K27, K29, K33, K48, K63, K48R and K63R) were kindly provided by Professor Chengjiang Gao. All constructs were confirmed by DNA sequencing. Plasmids were transiently transfected into HEK293T cells using the Lipofectamine 2000 reagent (Invitrogen) according to the manufacturer’s instructions.

### Coimmunoprecipitation assay

Whole-cell lysates were prepared in RIPA buffer with a protease inhibitor cocktail (Bimake, Texas, USA). After centrifugation at 13,000 rpm for 30 min, the supernatants were collected and incubated overnight at 4 ℃ with anti-Flag magnetic Beads (HY-K0201A, MCE, China). The beads were washed five times with lysis buffer. Eventually, immunoprecipitates were eluted by boiling with SDS sample buffer (Vazyme, China), and supernatants were analyzed by Western blotting.

### Ubiquitination assay

To analyze the ubiquitination of TRAF6 in HEK293T cells, the cells were transfected with plasmids expressing TRAF6-Flag, TRIM26-Myc, HA–ubiquitin (WT), as well as various HA–ubiquitin mutants (K6, K11, K27, K29, K33, K48, K63, or K48R). After transfection for 36 h, the whole cell extracts were immunoprecipitated with Flag magnetic beads and analyzed by immunoblotting with anti-HA antibodies.

### Immunofluorescence assay

HEK293T cells were seeded on 24-well slides and transfected with the indicated plasmids. After 24 h, the cells were fixed in 4% paraformaldehyde, permeabilized with 0.2% Triton X-100, and blocked with TBST containing 5% BSA. The cells were then washed and incubated with the indicated primary antibodies at 4 ℃ overnight, followed by incubation with Alexa Fluor 488-conjugated goat anti-mouse lgG and Alexa Fluor 594-conjugated goat anti-rabbit IgG secondary antibodies for 1 h at room temperature. Nuclei were counterstained with DAPI. Images were captured using a Zeiss LSM980 confocal microscope and processed with Zeiss Zen software.

### Flow cytometry

Mice were sacrificed at the indicated time after infection, and cells were collected by lavage with 1×PBS. Spleens were homogenized on ice, filtered through a cell strainer, and the cells collected by centrifugation at 1,000 rpm for 5 min. Cells were counted using a hemocytometer and stained with fluorochrome-coupled antibodies as indicated. Cell surface staining was performed for 30 min at 4 ℃. Intracellular staining was carried out using a fixation/permeabilization kit (Biolegend). Zombie dye was added to exclude dead cells. Stained cells were resuspended and subjected to flow cytometry. Data analysis was performed using CytExpert software.

### Histological analysis

Spleen samples were collected after infection and fixed in 10% neutral formaldehyde solution for 48 h. The fixed tissue was embedded in paraffin and cut into sections of 3 μm thickness. The tissue sections were stained with Hematoxylin and Eosin (H&E) according to the manufacturer’s instructions. Stained samples were observed using a light microscope (Olympus Optical Co., Tokyo, Japan).

### Statistical analysis

All experimental data were presented as means ± standard deviation, and were analyzed using GraphPad Prism 8 (San Diego, CA, USA). Two-tailed unpaired Student’s t-test was used for comparisons of two experimental groups, while one-way ANOVA with multiple-comparison correction was used for other analyses. All p-values were two-sided, and significance levels are indicated as * *p* < 0.05, ** *p* < 0.01, *** *p* < 0.001, **** *p* < 0.0001, respectively.

## Results

### *T. gondii* infection suppresses TRAF6 expression

**Fig. 1:**
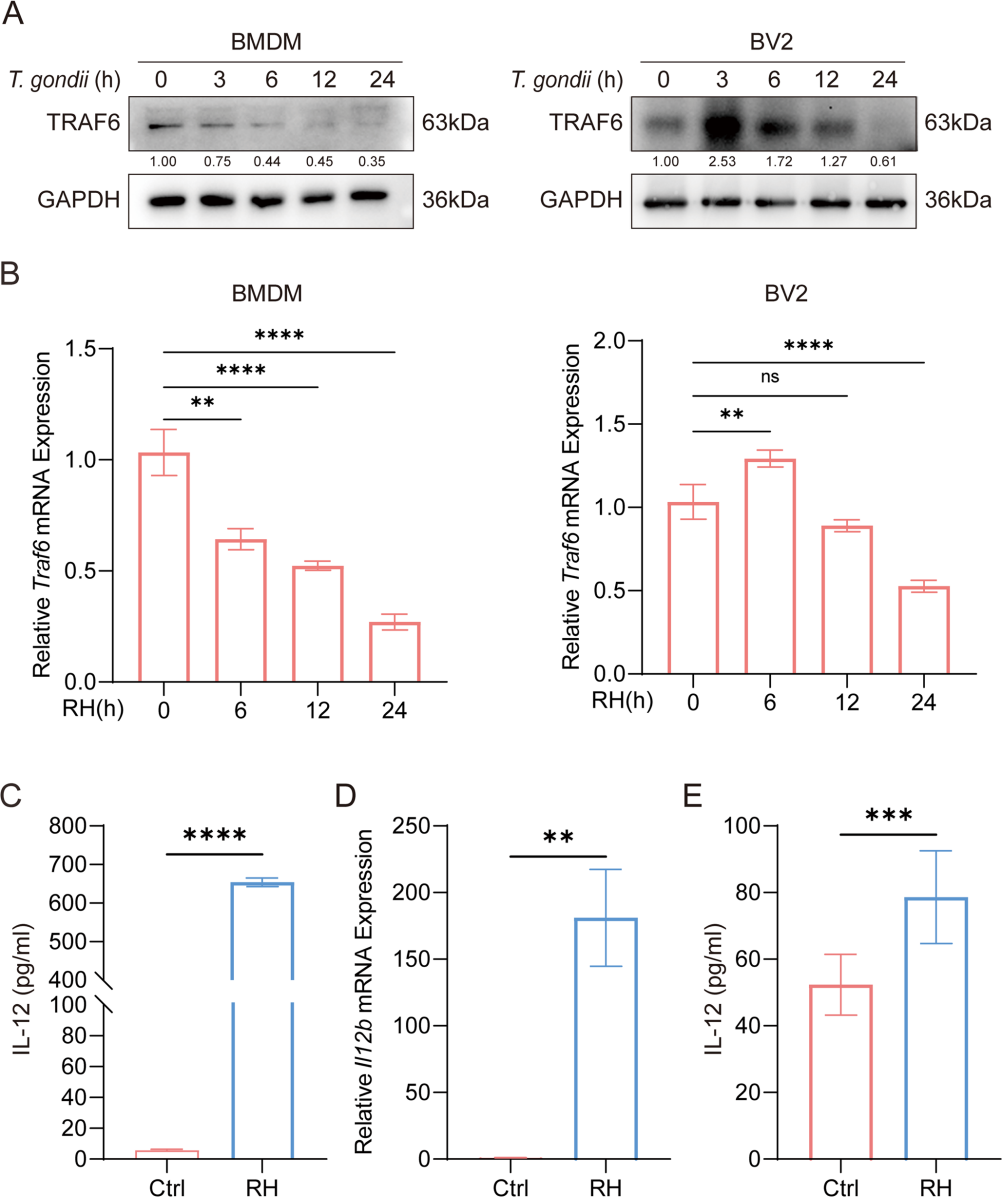
*T. gondii* infection downregulates TRAF6 expression and upregulates IL-12 expression. (**A**) *T. gondii* infection downregulated TRAF6 protein expression in bone marrow-derived macrophages (BMDMs) and BV2 microglial cells. (**B**) *T. gondii* infection downregulated TRAF6 mRNA expression in BMDMs and BV2 microglial cells. (**C**) BMDMs from WT mice were infected with *T. gondii* RH strain for 24 h (MOI = 3), and then enzyme-linked immunosorbent assay (ELISA) was conducted to assess the secretion levels of inflammatory cytokines IL-12. (**D**) BMDMs from WT mice were infected with *T. gondii* RH strain for 24 h (MOI = 3), and then qPCR was conducted to assess the mRNA transcription levels of inflammatory cytokines IL-12. (E) WT mice (*n* = 3 per group) were intraperitoneally infected with 100 RH strain tachyzoites and the serum level of IL-12 was measured three days post-infection (measured by ELISA). * *p* <0.05, ** *p* <0.01, *** *p* <0.001, **** *p* <0.0001; ns, not significant. Data are representative of three independent experiments

Given the established interaction between TRAF6 and the *T. gondii* virulence effector GRA7, which activates the MyD88 signaling pathway and promotes ROS production to restrict *T. gondii* infection [27, 28], we investigated whether TRAF6 expression is dynamically regulated in macrophages during *T. gondii* infection. We infected bone marrow-derived macrophages (BMDMs) and BV2 microglial cells with *T. gondii* RH strain for different time points, and then used Western blotting (WB) and quantitative real-time PCR (qPCR) to detect dynamic changes in TRAF6 protein and mRNA levels Fig. (1A and B). The results showed a significant, time-dependent reduction in TRAF6 expression following *T. gondii* infection, reaffirming TRAF6’s role in the host immune response against the parasite. To further explore the immune regulatory network associated with TRAF6 during infection, we examined the expression pattern of IL-12. In vitro experiments showed that BMDMs infected with *T. gondii* for 24 h exhibited marked upregulation of IL-12 mRNA transcription, and increased extracellular IL-12 secretion in cell culture supernatants (Fig. 1C and D). Consistent with these in vitro findings, ELISA analysis of serum from *T. gondii*-infected mice revealed a significant increase in IL-12 levels **(**Fig. 1E**)**. Collectively, these results demonstrate that TRAF6 is actively involved in the host anti-*T. gondii* immune response.

### TRIM26 binds to TRAF6

**Fig. 2:**
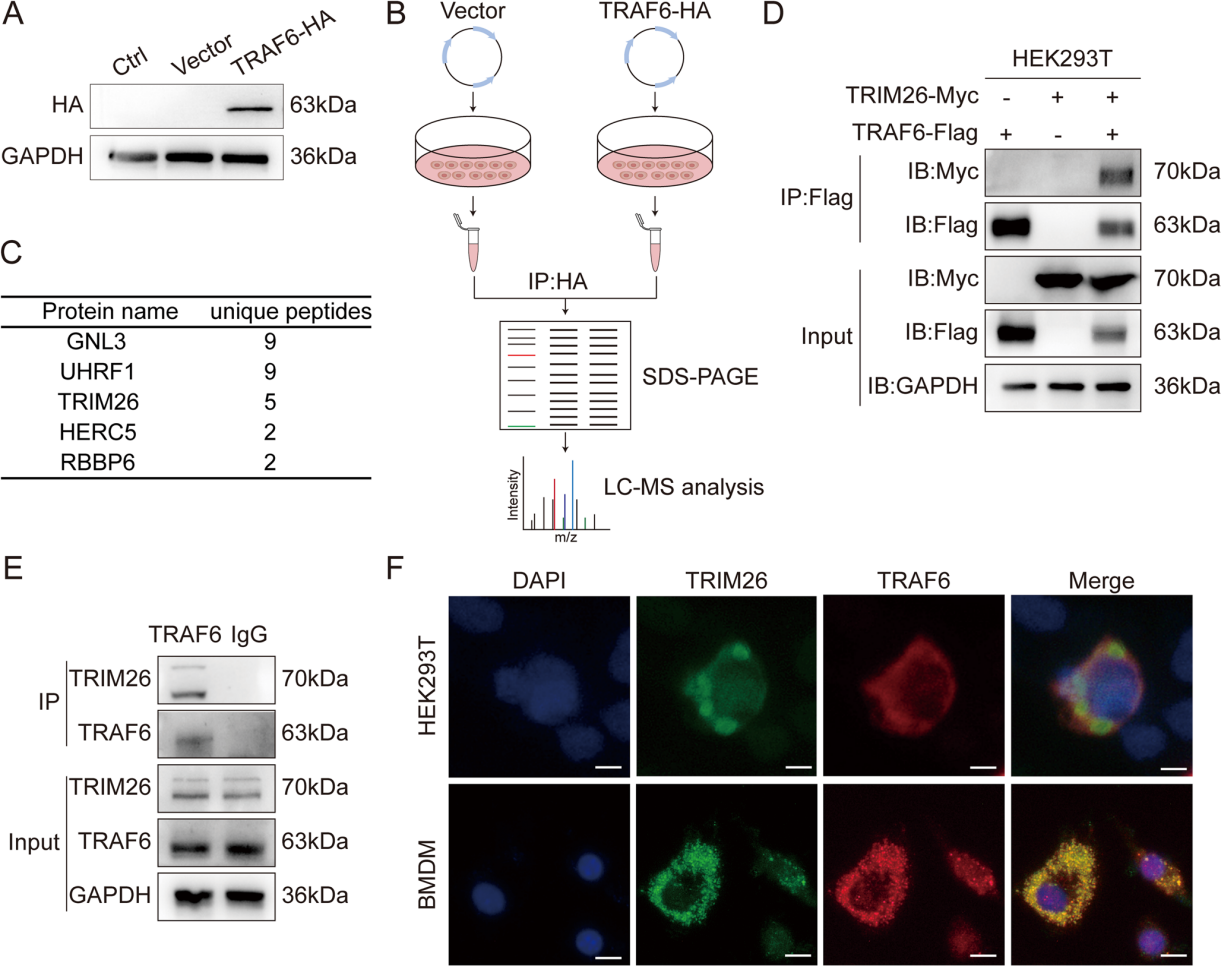
TRIM26 binds to TRAF6. (**A**) Verification of the overexpression efficiency of pcDNA3.1-TRAF6-HA plasmid by immunoblotting (IB). Ctrl: Untransfected HEK293T cells; Vector: HEK293T cells transfected with an empty vector (detection was performed using an anti-TRAF6 antibody). (**B**) Noninfected HEK293T cells were transfected with 3 µg of empty vector plasmid or pcDNA3.1-TRAF6-HA plasmid for 24 h. The TRAF6-containing complex was pulled down by immunoprecipitation (IP) using an anti-HA antibody. The immunoprecipitated products were subjected to SDS-PAGE, followed by liquid chromatography mass spectrometry (LC-MS) analysis. (**C**) Identification of protein names and unique peptides from the MS data. (**D**) HEK293T cells were transfected with the indicated plasmids. Cell lysates were subjected to IP with anti-Flag magnetic beads, followed by IB with anti-Flag and anti-Myc antibodies. (**E**) BMDMs were isolated from wild-type (WT) mice, cultured, and then used to prepare cell lysates. IP was performed using an anti-TRAF6 antibody or rabbit IgG antibody (as a negative control), and the immunoprecipitated products were analyzed by WB using anti-TRAF6 and anti-TRIM26 antibodies. (**F**) HEK293T cells transfected with TRIM26-Myc and TRAF6-Flag plasmids were fixed and stained with anti-Myc antibody (green) and an anti-Flag antibody (red). Additionally, BMDMs were stained with an anti-TRIM26 antibody (green) and an anti-TRAF6 antibody (red). The colocalization of TRIM26 and TRAF6 was visualized using confocal microscopy. Scale bars = 5 μm

To explore the regulatory mechanisms governing TRAF6 function, we first screened for proteins that interact with TRAF6. We constructed a TRAF6-HA expression plasmid and transfected it into HEK293T cells. The overexpression efficiency of TRAF6-HA was verified by WB **(**Fig. 2A**)**. Next, to identify potential TRAF6-interacting proteins, we performed immunoprecipitation (IP) using an anti-HA antibody to pull down the TRAF6-HA complex, followed by liquid chromatography-mass spectrometry (LC-MS) analysis. This screening identified several candidate interactors, including GNL3, UHRF1, TRIM26, HERC5, and RBBP6 (Fig. 2B and C). We then prioritized validating these protein-protein interactions. We constructed corresponding overexpression vectors for each candidate and performed co-immunoprecipitation (Co-IP) assays in HEK293T cells. Co-IP results showed that only TRIM26 was specifically co-precipitated with TRAF6 **(**Fig. 2D**)**. Furthermore, to confirm the endogenous interaction, we prepared lysates from BMDMs and performed IP using an anti-TRAF6 antibody. WB analysis of the precipitated complexes revealed that endogenous TRIM26 was specifically co-precipitated with TRAF6, confirming their native interaction (Fig. 2E). Finally, confocal microscopy analysis further demonstrated colocalization of TRIM26 and TRAF6 in HEK293T and BMDMs **(**Fig. 2F**)**. Collectively, these findings demonstrate that TRIM26 is a specific TRAF6-interacting protein.

### TRIM26 attenuates K48-linked polyubiquitination of TRAF6

**Fig. 3:**
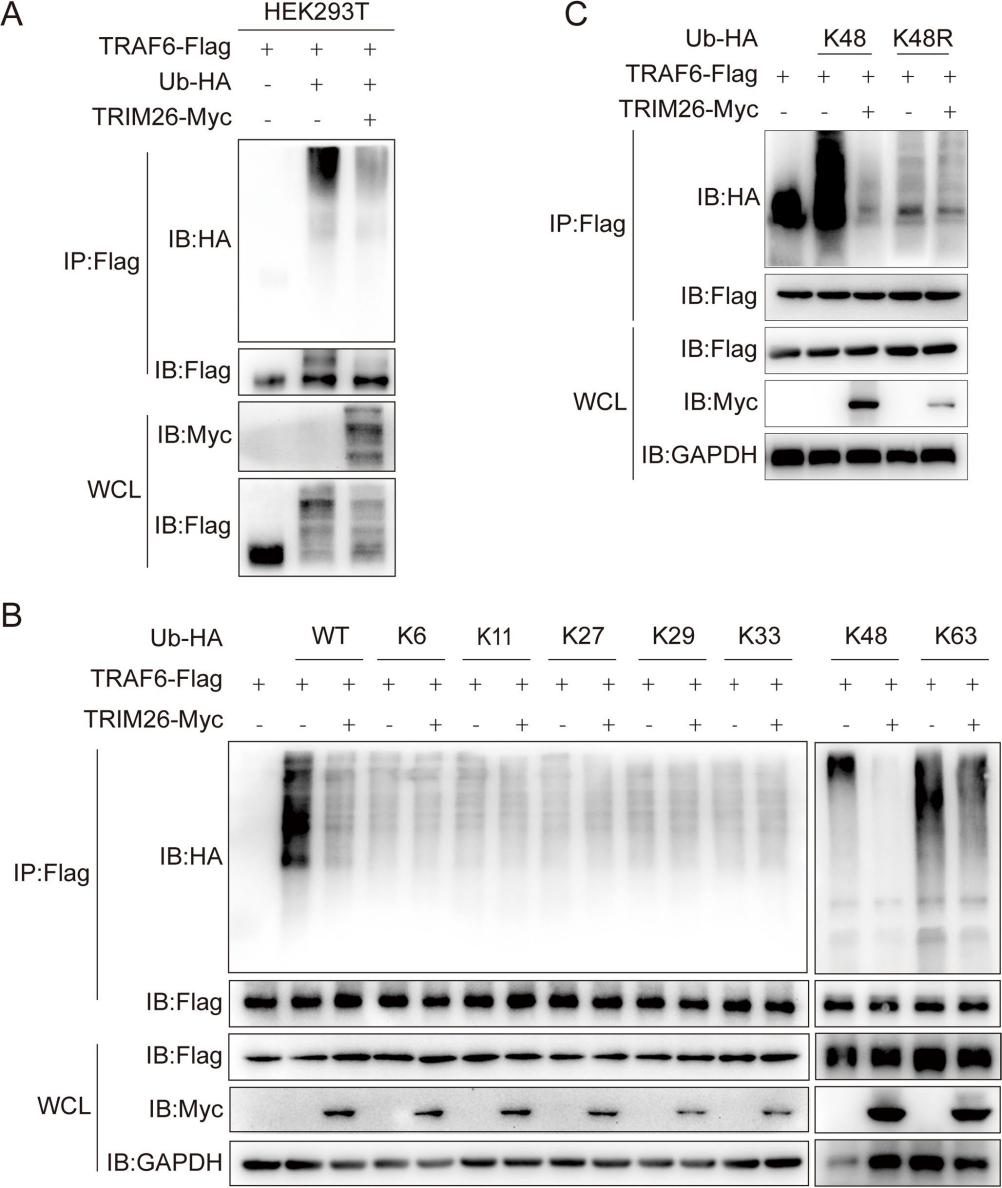
TRIM26 attenuates the K48-linked ubiquitination of TRAF6. (**A**) HEK293T cells were co-transfected with TRIM26-Myc, TRAF6-Flag, and WT-Ubiquitin-HA plasmids for 36 h. Whole cell lysates (WCL) were prepared, followed by IP against TRAF6 using anti-Flag magnetic beads. The ubiquitination level of TRAF6 was detected by WB with an anti-HA antibody. (**B**) HEK293T cells were co-transfected with TRIM26-Myc, TRAF6-Flag, WT Ubiquitin-HA and various single-lysine ubiquitin mutants (K6, K11, K27, K29, K33, K48, and K63) for 36 h. Cell lysates were subjected to IP with anti-Flag magnetic beads, and TRAF6 ubiquitination was analyzed by WB using an anti-HA antibody. (**C**) HEK293T cells were co-transfected with TRIM26-Myc, TRAF6-Flag, WT-Ub-HA, K48-Ub-HA and K48R-Ub-HA plasmids for 36 h. Cell lysates were subjected to IP with anti-Flag beads, followed by WB analysis with an anti-HA antibody

TRIM26, a member of the tripartite motif (TRIM) family, is known for its role in regulating substrate ubiquitination in anti-viral responses and tumor processes [29–31]. We hypothesized that TRIM26 might modulate TRAF6 ubiquitination. To test this, TRAF6-Flag, TRIM26-Myc, and HA-Ub-WT were overexpressed in HEK293T cells, and the immunoprecipitation assays were conducted. As shown in Fig. 3A, TRIM26 overexpression significantly reduced TRAF6 ubiquitination levels compared to the control group. Although TRIM26 is typically associated with the assembly of ubiquitin chains on substrates, its role in deubiquitination has been less explored. To elucidate the specific type of TRAF6 ubiquitination regulated by TRIM26, we co-transfected HEK293T cells with TRAF6-Flag, TRIM26-Myc, and a panel of ubiquitin mutants (K6, K11, K27, K29, K33, K48, K63)—each retaining only one intact lysine residue, with all other lysines substituted by arginine. IP assays revealed that TRIM26 significantly attenuated K48-linked polyubiquitination of TRAF6, while exerting no effect on other types of ubiquitination (Fig. 3B). This finding was further validated via transfection with a K48R-ubiquitin mutant, where TRIM26 failed to abrogate TRAF6 polyubiquitination, indicating that TRIM26 specifically targets K48-linked polyubiquitination **(**Fig. 3C**)**. Collectively, these results demonstrate that TRIM26 mediates the deubiquitination of TRAF6 by removing K48-linked polyubiquitination chains.

### TRIM26 positively regulates the NF-κB signaling pathway and promotes inflammatory cytokines production during *T. gondii* infection

**Fig. 4:**
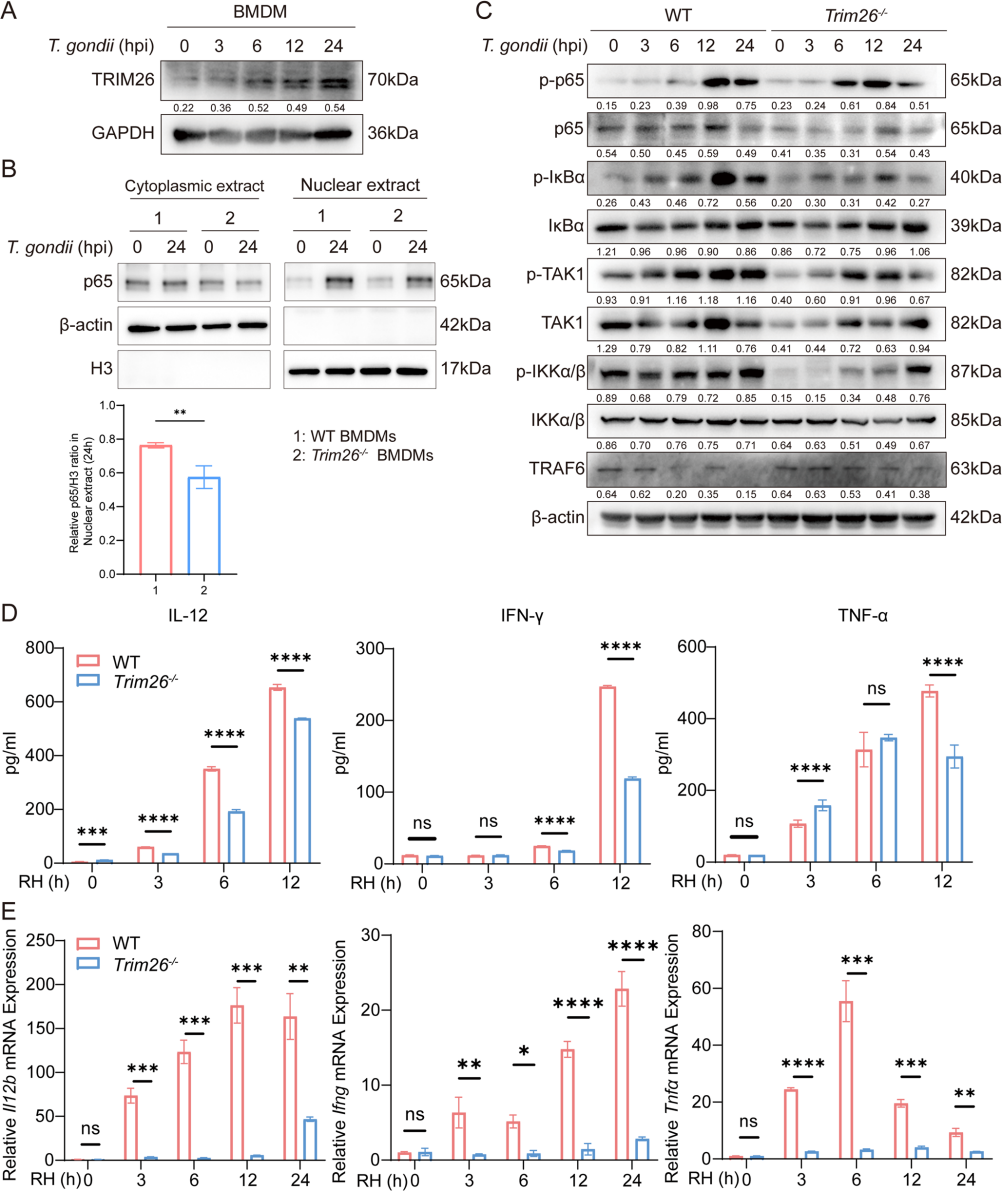
TRIM26 positively regulates the NF-κB signaling pathway and promotes inflammatory cytokine production during *T. gondii *infection. (**A**) BMDMs from WT mice were infected with *T. gondii* for 0, 3, 6, 12, and 24 h (MOI = 3). Cell lysates were collected, and WB was performed to detect changes in TRIM26 protein expression. (**B**) BMDMs were isolated from WT or *Trim26*^*−/−*^ mice. And BMDMs were infected with *T. gondii* for 0 h or 24 h (MOI = 3). Levels of p65 in the cytoplasmic and nuclear fractions were analyzed by WB analysis. (**C**) BMDMs from WT and *Trim26*^*−/−*^ mice were infected with *T. gondii* RH strain for 0, 3, 6, 12, and 24 h (MOI = 3). WB was conducted to detect the phosphorylation levels of NF-κB signaling-related proteins (p-p65, p-IκBα, p-TAK1, p-IKKα/β). (**D**) BMDMs from WT and *Trim26*^*−/−*^ mice were infected with *T. gondii* RH strain for 0, 3, 6, 12, and 24 h (MOI = 3). Enzyme-linked immunosorbent assay (ELISA) was used to determine the levels of inflammatory cytokines (IL-12, IFN-γ, and TNF-α) in cell culture supernatants. (**E**) BMDMs from WT and *Trim26*^*−/−*^ mice were infected with *T. gondii* RH strain for 0, 3, 6, 12, and 24 h (MOI = 3), and then qPCR was conducted to assess the mRNA transcriptional levels of inflammatory cytokines (IL-12, IFN-γ, and TNF-α). (* *p* < 0.05, ** *p* < 0.01, *** *p* < 0.001, **** *p* < 0.0001; ns, no significance)

Although TRIM26 has been studied in various contexts, its role in the inflammatory response to *T. gondii* infection remains uncharacterized [32, 33]. To clarify this, we infected BMDMs with *T. gondii* RH strain and analyzed TRIM26 expression via WB at indicated time points post infection. Results showed a time-dependent upregulation of TRIM26 during *T. gondii* infection (Fig. 4A).

To investigate how TRIM26 modulates cytokines production, we performed WB to analyze NF-κB signaling molecules in BMDMs from WT and *Trim26*^*−/−*^ mice. WB analysis of the cytoplasmic and nuclear fractions of WT and *Trim26*^*−/−*^ BMDMs revealed that after 24 h of stimulation, the nuclear translocation of NF-κB p65 in *Trim26*^*−/−*^ BMDMs was reduced **(**Fig. 4B**)**. Further analysis revealed decreased phosphorylation of MyD88-mediated signaling components upstream of p65, including IκBα, TAK1, and IKKα/β, in *Trim26*^*−/−*^ BMDMs. This indicates that TRIM26 is essential for activating the TRAF6-dependent NF-κB signaling pathway during *T. gondii* infection **(**Fig. 4C**)**. In vitro studies showed that infection of *Trim26*^*−/−*^ BMDMs with *T. gondii* RH strain resulted in significantly lower production of IL-12, IFN-γ, and TNF-α compared to WT controls (Fig. 4D). This reduction in cytokine levels was corroborated by decreased mRNA expression, as determined by quantitative real-time PCR assays **(**Fig. 4E**)**. Collectively, these findings demonstrate that TRIM26 promotes the production of pro-inflammatory cytokines by enhancing NF-κB signaling activity.

### TRIM26 is essential for the host immune response against *T. gondii *and the control of parasite expansion

**Fig. 5:**
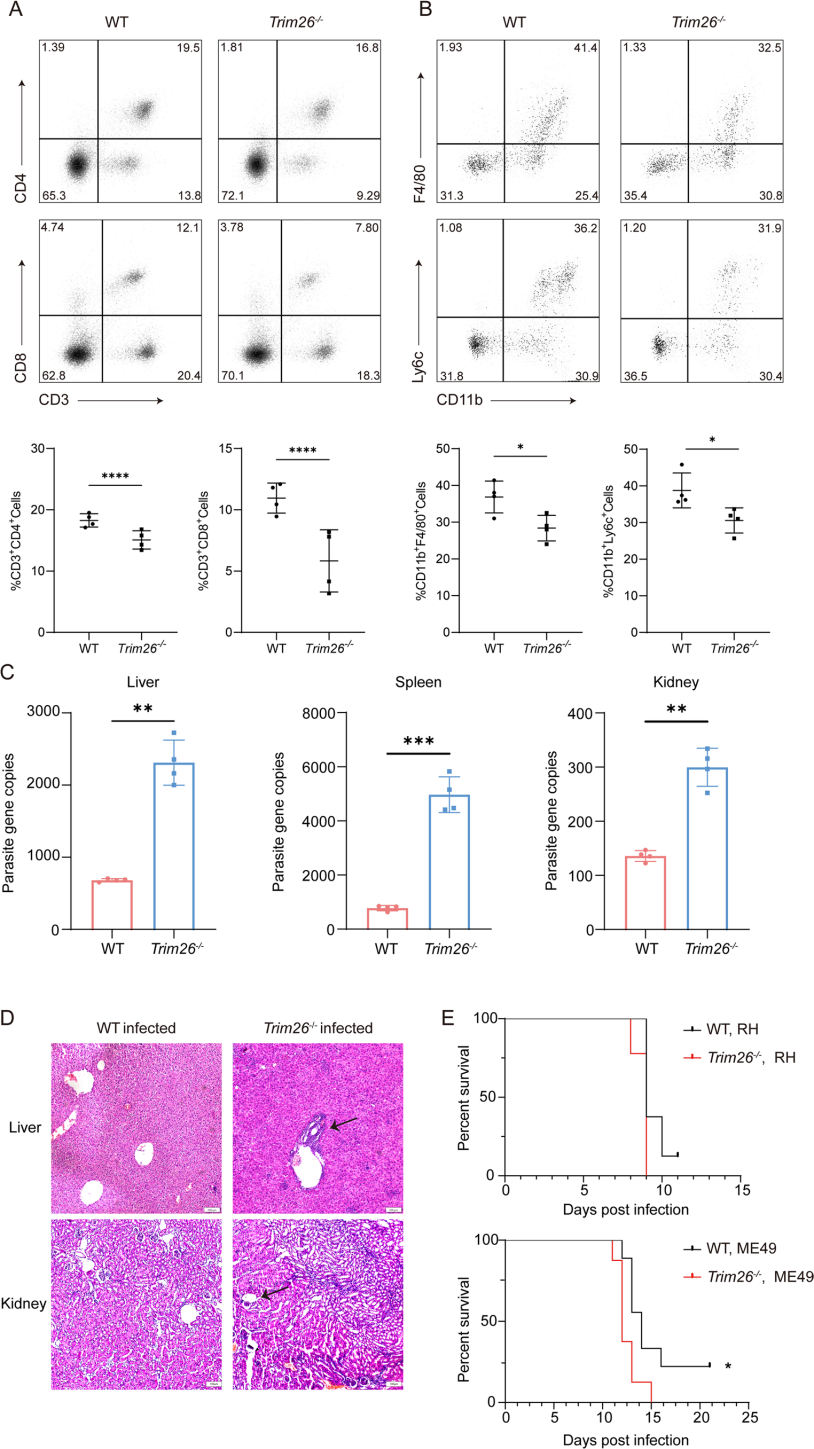
TRIM26 is essential for the host immune response against *T. gondii *and the control of parasite expansion. (**A**) WT and *Trim26*^*−/−*^ mice were intraperitoneally infected with 100 RH strain tachyzoites for 8 days, after which spleens were harvested and disrupted to prepare single-cell suspensions. The cells were stained with fluorescently labeled antibodies specific for CD45, CD3, CD4, and CD8, followed by flow cytometry analysis to determine the proportions of CD4^+^ and CD8^+^ T cells. The percentage of CD4^+^ and CD8^+^ cells were quantified via flow cytometry. Mean percentages ± SD were calculated from at least three independent experiments. (**B**) WT and *Trim26*^*−/−*^ mice were intraperitoneally infected with 100 RH strain tachyzoites for 8 days, after which ascites were collected and processed into single-cell suspensions. The cells were stained with fluorescently labeled antibodies specific for CD45, CD11b, F4/80, and Ly6c. Flow cytometry was performed to determine the proportions of macrophages (CD11b^+^F4/80^+^) and inflammatory monocytes (CD11b^+^Ly6c^+^). The percentage of CD11b^+^F4/80^+^ and CD11b^+^Ly6c^+^ cells were quantified via flow cytometry. Mean percentages ± SD were calculated from at least three independent experiments. (**C**) WT and *Trim26*^*−/−*^ mice were intraperitoneally infected with 100 RH strain tachyzoites for 6 days, after which spleens, livers, and kidneys were harvested to extract genomic DNA. Absolute quantification PCR was used to determine the absolute copy number of *T. gondii* gDNA in these tissues. (**D**) Livers and kidneys were collected from *T. gondii*-infected WT and *Trim26*^*−/−*^ mice, and tissue sections were prepared. The sections were stained with hematoxylin and eosin (H&E) staining to assess histopathological changes induced by *T. gondii* infection. (**E**) WT and *Trim26*^*−/−*^ mice (*n* = 9 per group) were infected with 100 tachyzoites of the *T. gondii* ME49 or RH strain, and survival was monitored daily for the indicated period. (* *p* < 0.05, ** *p* < 0.01, *** *p* < 0.001, **** *p* < 0.0001; ns, no significance)

To evaluate the functional role of TRIM26 in the in vivo immune response to *T. gondii* infection, we induced toxoplasmosis in *Trim26*^*−/−*^ and WT mice. Given that macrophages and inflammatory monocytes are sources of IL-12, and T cells are the primary producers of IFN-γ in the spleen [10, 34, 35], we investigated how TRIM26 regulates immune cell proportions. Mice were infected intraperitoneally with 100 *T. gondii* RH strain tachyzoites, and peritoneal cavity cells were analyzed at 8 days post-infection. Compared to WT controls, *Trim26*^*−/−*^ mice exhibited significantly reduced proportions of CD4^+^ and CD8^+^ T cells **(**Fig. 5A**)**. Additionally, the numbers of macrophages and inflammatory monocytes were also reduced in *Trim26*^*−/−*^ mice following infection **(**Fig. 5B**)**. These findings highlight the pivotal role of TRIM26 in driving immune signaling pathway in vivo during *T. gondii* infection.

To determine whether impaired immune responses in *Trim26*^*−/−*^ cells affected parasite control, we infected *Trim26*^*−/−*^ and WT mice with *T. gondii* RH tachyzoites and measured parasite loads in multiple organs. Quantitative RT-PCR analysis revealed elevated levels of *T. gondii* genomic DNA in the liver, spleen, and kidney of *Trim26*^*−/−*^ mice compared to WT controls (Fig. 5C). Histopathological examination further confirmed more severe injury in the liver and kidney of *Trim26*^*−/−*^ mice after *T. gondii* infection **(**Fig. 5D**)**. Furthermore, *Trim26*^*−/−*^ mice displayed significantly higher susceptibility to challenge with the ME49 strain, though not with the highly virulent RH strain, compared to *Trim26*^*−/−*^ mice **(**Fig. 5E**)**. These findings demonstrate that the elevated parasite burdens and increased mortality in *Trim26*^*−/−*^ mice result from impaired immune responses, which fail to restrict parasite expansion during toxoplasmosis. Thus, our findings indicate that TRIM26 is crucial for mediating the host immune response against *T. gondii* and controlling infection.

## Discussion

The innate immune system serves as the primary defense against pathogenic organisms and plays a crucial role in mounting an immune response to *T. gondii* infection. Understanding the interaction between *T. gondii* and the host immune system is vital for developing effective therapeutic strategies. This study presents evidences that TRIM26 can regulate the immune response to *T. gondii* by modulating TRAF6 K48-linked polyubiquitination, which enhances NF-κB pathway activity and elevates cytokines levels, ultimately influencing outcome of toxoplasmosis. Our findings reveal TRIM26 as a novel regulator of TRAF6 ubiquitination and highlight its role in the host immune network.

TRAF6, an adaptor protein in the NF-κB pathway, has been extensively studied in cancer, inflammation and cell development [24, 36–39]. Previous studies have shown that TRAF6 directly targets *T. gondii* nanovesicles, in conjunction with TRIM21, enhancing the accumulation of downstream ubiquitin-related proteins and activating intracellular autophagosomes and inflammasomes, ultimately leading to the destruction of the nanovesicles [40]. Additionally, GRA15, a *T. gondii*-derived protein, has been shown to physically and functionally interact with TRAF2 and TRAF6, activating the NF-κB pathway and enhancing the host’s protective immune response during *T. gondii* infection [41]. This accumulating evidence positions TRAF6 as a critical regulator in toxoplasmosis, although the internal regulatory mechanisms governing TRAF6 modification during *T. gondii* infection remain poorly understood. Our study elucidates the regulatory relationship between TRAF6 and interacting proteins, offering new insights into its protein modification mechanism, and providing a potential target for controlling *T. gondii* infection.

We demonstrated that TRAF6 protein stability is dynamically regulated in murine macrophages, with expression levels decreasing over time in BMDMs, but it first increases and then decreases in BV2 cells. The intriguing divergent expression patterns of TRAF6 observed between these two cell lines may be attributed to their distinct cellular origins and tissue-specific contexts. Specifically, the BV2 microglial cell line is derived from and models brain-resident microglia. The precise molecular mechanisms underlying these differences require further experimental investigation to be fully elucidated. The *T. gondii* infection also elevates IL-12 and IFN-γ levels in vivo and in vitro. Further mechanistic investigations revealed that TRIM26, a novel TRAF6-interacting protein, attenuated K48-linked polyubiquitination, a modification commonly associated with proteasomal degradation. TRIM26 has been extensively investigated in the context of cancer, autophagy and microbial infections, where it has been shown to regulate immune responses. For example, TRIM26 can bind to IRF3 within the nucleus, promoting its degradation. This negative regulation ultimately reduces IFN-β production and diminishes antiviral capabilities [42]. In sepsis and enteritis disease models, TRIM26 mediated K11 ubiquitination of TAB1 enhancing TAK1 phosphorylation and positively regulating downstream signal transduction and proinflammatory cytokine secretion [43]. Additionally, TRIM26 exerts antifungal immunity through restricting neutrophil activity and limiting proinflammatory cytokines expression during *Candida* infection [33]. However, the role of TRIM26 in *T. gondii* infection has not been previously elucidated. Our animal experiments indicated that *Trim26*^*−/−*^ mice were significantly more susceptible to toxoplasmosis, exhibiting earlier onset, more severe symptoms, and increased parasite loads in the liver, kidney, and spleen, accompanied more pronounced pathological tissue damage in these organs. These findings indicate that TRIM26 is critical for the host’s resistance to toxoplasmosis, as its deficiency impairs the ability to control of parasite invasion and proliferation.

Our findings align with previous reports on the role of ubiquitination in immune regulation during *T. gondii* infection. For instance, RNF213, an E3 ubiquitin ligase, has been shown to mediate IFN-γ control of *T. gondii* by promoting the ubiquitination of nanovesicles, restricting the parasite’s growth [44]. Similarly, OTUD1, a deubiquitinating enzyme, stabilize UBC13 protein expression and enhance the ubiquitination of IRAK1 and TRAF6 in *T. gondii*-infected dendritic cells. This process activates the NF-κB pathway and ultimately increases IL-6, IL-12 and TNF-α expression [45].

Interestingly, we found that TRIM26 overexpression reduces TRAF6 ubiquitination, particularly K48-linked polyubiquitination, which challenges the conventional understanding of E3 ubiquitin ligase function. Similar findings were reported by Mahlokozera et al. who demonstrated that TRIM26 interacts with SOX2 and reduce its ubiquitination, thereby stabilize SOX2 protein levels [46]. These observations support our results and suggest that TRIM26 possesses diverse biological roles beyond its function as an E3 ligase.

It’s noteworthy that K48-linked ubiquitination determines the stability of proteins. As TRAF6 occupies an important position in immune network, particularly in the NF-κB pathway, which masters the expression levels of IL-12 and IFN-γ, further research is imperative to deeply understand the mechanism of TRIM26 in *T. gondii* infection. Our findings reveal that TRIM26 can enhance the stability of TRAF6 protein by preventing TRAF6 from proteasome degradation, thereby promoting NF-κB p65 nuclear translocation. This process enhances NF-κB pathway activity and the expression of downstream proinflammatory cytokines. In contrast, TRIM26 could not affect the K63-linked ubiquitination of TRAF6, which is a form of auto-ubiquitination, illustrating that TRIM26 modulates the protein level of TRAF6 rather than its biological function. However, previous studies have indicated that TRIM26 can also regulate TAK1 activity by mediating the ubiquitination of TAB1, thereby promoting the activation of the NF-κB pathway [43]. Thus, TRIM26 exerts a clear positive regulatory effect on the NF-κB signaling pathway. Its mechanisms of action are diverse and not reliant on a single pathway, reflecting the functional complexity of TRIM26.

Furthermore, the *T. gondii* protein GRA15 has been demonstrated to serve as an initiator for activating the host NF-κB pathway. Type II strains lacking GRA15 exhibit severe defects in both NF-κB nuclear translocation and NF-κB-mediated transcription [47]. Moreover, GRA15 can recruit ubiquitin ligases, including TRAF2 and TRAF6, to the vacuolar membrane, thereby enhancing the recruitment of ubiquitin receptors (p62/NDP52) and ubiquitin-like molecules (LC3B and GABARAP). This process ultimately leads to the lysosomal degradation of the vacuole [19]. In addition, by binding to TRAF6, GRA15 promotes the mouse type I interferon response through mediating STING polyubiquitination and enhancing cGAS/STING signaling [48].

Taken together, the observed detrimental effects of GRA15 on the parasite might suggest that possessing GRA15 appears disadvantageous for *T. gondii*. However, excessive virulence leading to rapid host cell death would not benefit parasite survival. Therefore, to enhance transmission probability, *T. gondii* needs to balance immune evasion—to facilitate replication and dissemination—with appropriate immune activation to prevent premature host death. GRA15 appears to be an effector that attenuates parasite virulence and aids host survival.

Our results similarly highlight the important role of TRAF6 in activating the host NF-κB pathway upon *T. gondii* infection, a process regulated by TRIM26. Whether GRA15 is involved in this mechanism remains an open question worthy of further investigation. Addressing this would not only clarify the significant function of GRA15 but also provide new insights into the interaction mechanisms between *T. gondii* and host.

Despite the insights gained from our study, certain limitations need to be addressed in future research. A detailed exploration of the specific mechanism by which TRIM26 modulates TRAF6 ubiquitination is essential. Although we demonstrated that TRIM26 attenuates K48-linked ubiquitination, the precise molecular interactions involved in this process remain unclear. Furthermore, while we focused on the role of TRIM26 in modulating TRAF6 stability, its potential interactions with other components of the ubiquitination machinery, such as deubiquitinases or other E3 ligases, should be investigated. In addition, the role of *T. gondii* infection in the process of TRIM26-mediated deubiquitination of TRAF6 should be assessed. Understanding how *T. gondii* manipulates TRIM26 expression could provide valuable insights into the parasite’s immune evasion strategies. We also need to assess the activation level of DCs, as they are the primary cellular source of IL-12 secretion during *T. gondii* infection.

**Fig. 6:**
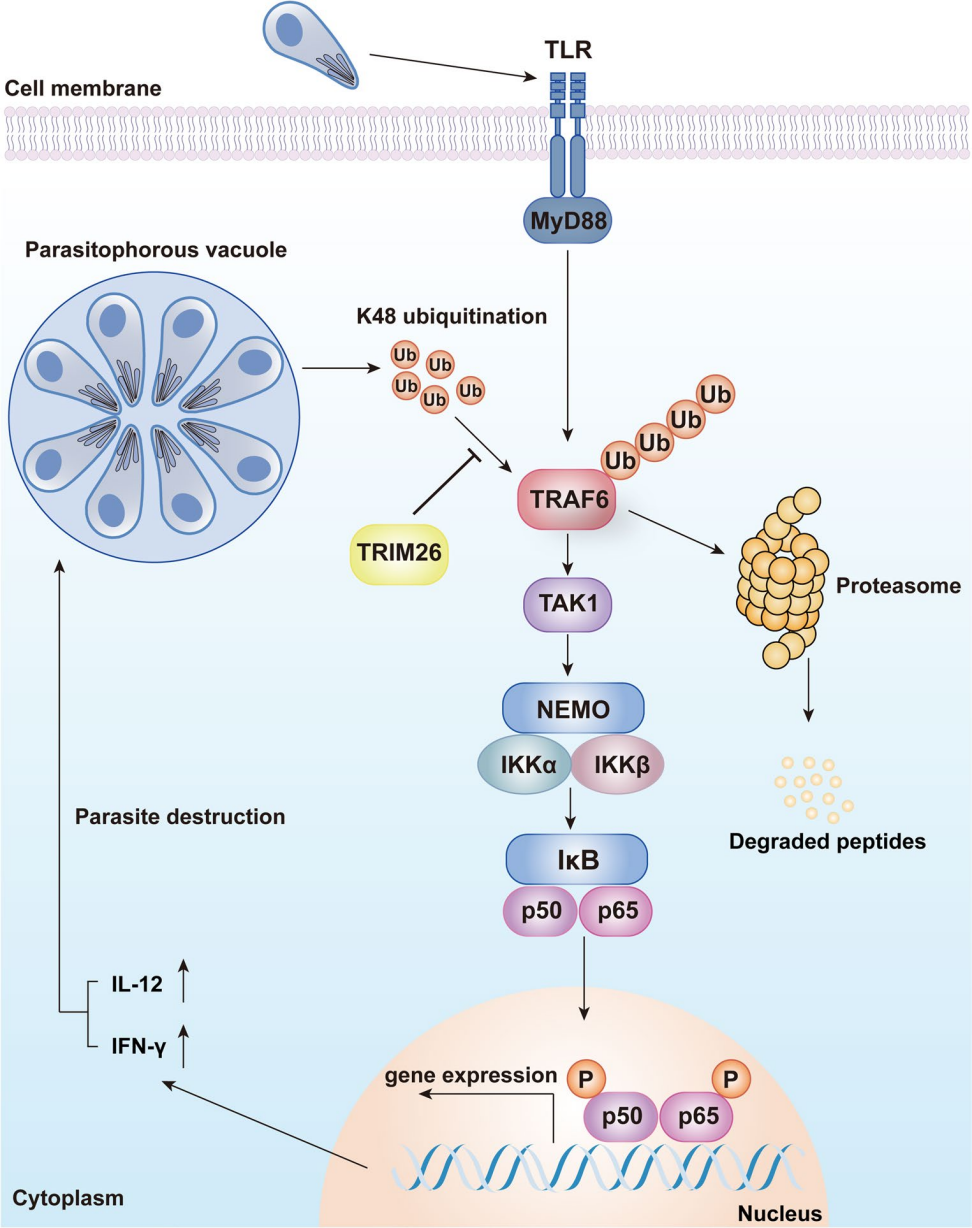
Schematic model of TRIM26 regulating the host immune response against *T. gondii *infection. A proposed model illustrating how TRIM26 mediates deubiquitination of TRAF6 by specifically antagonizing its K48-linked ubiquitination. This stabilization of TRAF6 facilitates the activation of the NF-κB signaling pathway and the subsequent production of pro-inflammatory cytokines

In summary, our study highlights the pivotal role of TRIM26 in regulating TRAF6 ubiquitination and its significance in the immune response against *T. gondii*, thereby proposing a novel target and concept for the prevention and treatment of *T. gondii* infection. By modulating TRAF6 K48-linked polyubiquitination, TRIM26 enhances NF-κB pathway activity and promotes the expression of proinflammatory cytokines (Fig. 6). These findings suggest that TRIM26 may serve as a novel therapeutic target for the prevention and treatment of toxoplasmosis. Further research should aim to fully elucidate the molecular mechanisms involved and to explore the broader implications of TRIM26 regulation in host-pathogen interactions.

## Supplementary Information

Below is the link to the electronic supplementary material.

ESM 1 (XLSX 11.0 KB)

## Data Availability

The datasets supporting the findings of this study are available within the article and its Supplementary Materials.
